# Sex Differences in Medium Spiny Neuron Excitability and Glutamatergic Synaptic Input: Heterogeneity Across Striatal Regions and Evidence for Estradiol-Dependent Sexual Differentiation

**DOI:** 10.3389/fendo.2018.00173

**Published:** 2018-04-18

**Authors:** Jinyan Cao, Jaime A. Willett, David M. Dorris, John Meitzen

**Affiliations:** ^1^Department of Biological Sciences, North Carolina State University, Raleigh, NC, United States; ^2^W.M. Keck Center for Behavioral Biology, North Carolina State University, Raleigh, NC, United States; ^3^Graduate Program in Physiology, North Carolina State University, Raleigh, NC, United States; ^4^Center for Human Health and the Environment, North Carolina State University, Raleigh, NC, United States; ^5^Comparative Medicine Institute, North Carolina State University, Raleigh, NC, United States

**Keywords:** sex, estradiol, hormones, striatum, medium spiny neuron, nucleus accumbens, caudate–putamen, electrophysiology

## Abstract

Steroid sex hormones and biological sex influence how the brain regulates motivated behavior, reward, and sensorimotor function in both normal and pathological contexts. Investigations into the underlying neural mechanisms have targeted the striatal brain regions, including the caudate–putamen, nucleus accumbens core (AcbC), and shell. These brain regions are of particular interest to neuroendocrinologists given that they express membrane-associated but not nuclear estrogen receptors, and also the well-established role of the sex steroid hormone 17β-estradiol (estradiol) in modulating striatal dopamine systems. Indeed, output neurons of the striatum, the medium spiny neurons (MSNs), exhibit estradiol sensitivity and sex differences in electrophysiological properties. Here, we review sex differences in rat MSN glutamatergic synaptic input and intrinsic excitability across striatal regions, including evidence for estradiol-mediated sexual differentiation in the nucleus AcbC. In prepubertal animals, female MSNs in the caudate–putamen exhibit a greater intrinsic excitability relative to male MSNs, but no sex differences are detected in excitatory synaptic input. Alternatively, female MSNs in the nucleus AcbC exhibit increased excitatory synaptic input relative to male MSNs, but no sex differences in intrinsic excitability were detected. Increased excitatory synaptic input onto female MSNs in the nucleus AcbC is abolished after masculinizing estradiol or testosterone exposure during the neonatal critical period. No sex differences are detected in MSNs in prepubertal nucleus accumbens shell. Thus, despite possessing the same neuron type, striatal regions exhibit heterogeneity in sex differences in MSN electrophysiological properties, which likely contribute to the sex differences observed in striatal function.

## Introduction

Steroid sex hormones and biological sex are important factors influencing neuron function (1–4). Historically, research into the roles of biological sex and hormones targeted brain regions directly involved in sex-specific reproduction-related behaviors in adult animals (5, 6). These regions display large sex differences in volume, neuron cellular anatomy, and/or electrophysiological properties. Examples of these regions include the spinal nucleus of the bulbocavernosus (7), the sexually dimorphic nucleus of the preoptic area (8), and the song control nuclei in sexually dimorphic songbirds (9). Years of work have built upon these early studies to make significant advances. Notable among these is the discovery that biological sex and steroid sex hormones can modulate brain regions not directly involved with sex-specific reproductive behaviors. Although the extent of the influence on biological sex in the nervous system remains vastly underexplored, especially since few studies documented experimental animal sex (10–12), contemporary research has detected sex differences in neural substrates across the brain (13–18). The newly appreciated role of sex in modulating neural substrate also includes the striatal brain regions, comprising the caudate–putamen (also called dorsal striatum), the nucleus accumbens core (AcbC) and the nucleus accumbens shell (AcbS).

Biological sex, exogenous 17β-estradiol (estradiol), and endogenously circulating hormones *via* the estrous or menstrual cycle can modulate striatal-mediated cognitive, locomotor, and sensorimotor behaviors, including those related to motivation and reward (19–30). Many striatal pathologies are sensitive to estradiol and/or show sex differences in incidence and/or phenotype. These include depression, Parkinson’s disease, drug addiction, schizophrenia, tardive dyskinesia, Huntington’s disease, ADHD, and Tourette’s syndrome, among others (31–41). The majority of published research in this field probes the link between sex and estradiol-induced influences on striatal-mediated normal and pathological behaviors (20, 42–46), especially regarding critical neurotransmitter/modulator systems such as dopamine and acetylcholine (43, 47–64). This research has established that sex and estradiol can influence striatal function *via* action on neurotransmitter and modulator systems, especially since the striatal regions show no robust sex differences in regional volume, neuron density, or soma size (65, 66). One area that has historically received less attention is how sex and estradiol modulate the electrophysiological properties of striatal neurons, including both the output neuron of the striatum, the medium spiny neuron (MSN) (67), and striatal interneurons (68). The term MSN is synonymous with striatal projection neuron. This is unfortunate, given that to change striatal circuit output and ultimately function, estradiol and sex must in some respect influence the electrophysiology of the MSN. This mini-review will focus upon the current state of knowledge regarding sex differences in rat MSN electrophysiological properties across striatal regions, with a focus on glutamatergic inputs and intrinsic excitability.

## MSNs in Adult Caudate–Putamen Show Sex-Specific and Estradiol-Induced Differences in Excitability *In Vivo*

This research began in the 1980s, when Vincent and colleagues discovered that estradiol exposure increased *in vivo* spontaneous action potential generation and dopamine sensitivity in striatal neurons in ovariectomized adult female rat caudate–putamen (69). Tansey and colleagues then identified that the striatal neurons showing increased *in vivo* spontaneous action potential generation in response to high estradiol levels included nigrostriatal MSNs (other MSN subtypes and striatal interneurons were not examined). This increase in spontaneous action potential generation was induced either *via* exogenous estradiol exposure in ovariectomized animals or endogenously during specific phases of the estrous cycle (70). MSN spontaneous action potential firing rates were elevated in females compared with males only during the phases of the estrous cycle associated with the effects of increased estradiol and progesterone (proestrus and estrus). MSNs outside of the caudate–putamen were not examined in either study. The next breakthrough in targeting MSN electrophysiology came in the mid-1990s, when Mermelstein and colleagues established that estradiol rapidly decreases L-type calcium channel currents in both prepubertal and adult female rat caudate–putamen MSNs (71). In this case, estradiol acted within seconds in a steroid- and dose-dependent method on a membrane-associated estrogen receptor. The receptor was eventually identified as membrane-associated estrogen receptor β (72). Later research encompassing both the caudate–putamen and the nucleus accumbens established the presence of membrane-associated estrogen receptors α, β, and GPER-1 in MSNs, striatal interneurons, presynaptic terminals, and glia (72–78). The presence of aromatase, the enzyme that converts testosterone into estradiol, was also confirmed (79–82). There is little to no evidence of nuclear estrogen receptors in the striatal regions in adult rodents (83–85), although an exhaustive search across development, estrus cycle stages, and relevant species has not been performed.

These studies established several foundational themes for more recent research on the influences of estradiol and sex on MSN electrophysiology. First, estradiol can act *directly* on MSNs to modulate electrophysiological properties in addition to *indirectly* acting on MSNs by manipulating neuromodulatory influences such as those encompassed by the dopaminergic and cholinergic systems. Second, MSN sensitivity to estradiol can occur in a sex-specific fashion. Third, estradiol can manipulate MSN excitability, and in particular can increase MSN excitability in adult female animals. This sex-specific increase in MSN excitability could be potentially induced by multiple cellular mechanisms, broadly grouped into two types: mechanisms inducing alterations in synaptic input onto MSNs and mechanisms inducing alterations in the intrinsic electrophysiological properties of MSN. These mechanisms are not necessarily mutually exclusive and are not necessarily active in every striatal region. Recent research has uncovered that both mechanism types can influence MSN excitability, with heterogeneity across striatal regions (Table 1).

**Table 1:** Development of sex differences in MSN electrophysiological properties varies by striatal region.

Electrophysiological property	Developmental stage	Caudate–putamen	Nucleus Accumbens Core	Nucleus Accumbens Shell
Intrinsic neuronal excitability	Prepubertal	Female > male	Female = male	Female = male
	Adult	?^a^	?	?

Excitatory synaptic Input	Prepubertal	Female = male	Female > male	Female = male
	Adult	?^a^	Female > male^b^	Female = male?^b^^,^^c^

*^a^Adult caudate–putamen medium spiny neurons (MSNs) show increased excitability *in vivo* in females compared with males, but the mechanisms underlying this phenomenon remains unknown and are not included in this table*.

*^b^Animals were gonad intact, but female estrous cycle stage has not been comprehensively examined*.

*^c^Most but not all accumbens shell (AcbS) literature shows no evidence of sex differences or estrogen sensitivity in excitatory synaptic input in adult animals unexposed to adverse environmental stimuli*.

*?—Data not available or complex*.

*Color signifies the presence of a sex difference*.

## Excitatory Synapse Number is Increased Onto Female Compared with Male AcbC MSNs, and These Synapses are Modulated by Estradiol in Adulthood

Regarding the AcbC, in the early part of this decade, Woolley and colleagues formulated the hypothesis that excitatory synapses onto MSNs in this region are increased in females compared with males (86). Anatomical studies employing electron microscopy, immunocytochemistry, and other techniques established that increased excitatory synapses are present on MSNs in the AcbC of adult rat females in proestrus compared with gonad-intact males (86–88). Alterations in excitatory synapse activity instruct AcbC function in many contexts (89–93), including the responsiveness to drugs of abuse (94). Even minor sex differences in these excitatory synaptic inputs are potentially influential on AcbC function.

One neuroanatomical correlate of increased excitatory synapse number onto female compared with male rat AcbC MSNs was concomitant increased dendritic spine density (87, 88). This sex difference in dendritic spine density was also identified in adult human AcbC, with increased dendritic spine density on female compared with male MSNs (95). In other brain regions, dendritic spine density has long been documented to be sensitive to estradiol exposure, either *via* endogenous exposure *via* the estrous or menstrual cycles, or exogenous (96–98). Regarding the AcbC, Staffend et al. elucidated that a 2-day exposure to exogenous estradiol altered dendritic spine density on MSNs in gonadectomized female hamsters (99). This exogenous estradiol-induced change in spine density is also detected in gonadectomized rat females and is dependent upon estradiol activating mGluR5 and endocannabinoid signaling *via* CB1 receptors (100, 101). This estrogen receptor/mGluR5 signaling pathway in the AcbC is speculated to induce an increased drive for sex (23), although this has not been tested in the context of the estrous cycle. Regarding neurological disorders, this pathway is implicated as one mechanism underlying estradiol-induced potentiation of cocaine-induced locomotor sensitization and cocaine self-administration (102, 103). This estradiol-induced change in dendritic spine density seems specific to the AcbC, at least in the absence of other interacting variables. No estradiol-induced changes in dendritic spine density were measured in the caudate–putamen, and only one of three experiments showed an estradiol-induced change in spine density in the AcbS (99, 101). There is select evidence that postsynaptic excitatory synapse markers are also increased in female AcbS (86), but this is not a robust finding (88, 104). There is evidence that sex differences in excitatory synapse in the AcbS are induced by the effects of stress and potentially other environmental factors (105). In AcbS MSNs, a stress paradigm induced sex-specific alterations in presynaptic but not postsynaptic excitatory synapse markers (106). Investigating the interactions between sex, hormones, and environmental inputs such as stress or environmental chemical exposure is an essential future line of research. Regarding male MSNs, another possibility is that testosterone regulates excitatory synaptic input onto nucleus accumbens neurons, as suggested by experiments analyzing dendritic spine density in response to week-long exogenous testosterone exposure in gonad-intact male rats (107), and the role of androgens in reward-related behaviors (108).

## Increased Excitatory Synapse Activity in Female AcbC MSNs is Present Before Puberty and is Blocked by Neonatal Exposure to Estradiol

This body of data indicates that excitatory synapse number is increased onto adult female compared with male MSNs in the AcbC. Whether these differences in excitatory synapse number are functional has been assessed by analyzing miniature excitatory postsynaptic current (mEPSC) properties (Table 1). Wissman and colleagues discovered increased mEPSC frequency in adult gonad-intact female compare to male rat AcbC MSN, with estrous cycle not formally assessed (88). No differences were detected in mEPSC amplitude, decay, or in paired pulse properties, consistent with a model of increased excitatory synapse number in female compared with male AcbC, although sex differences in synaptic release probability remain possible. This increase in excitatory synaptic input could potentially be generated either in puberty or during the early natal sensitive period, and then modulated in adult females by the estrous cycle. Cao and coauthors addressed this question in three respects (109). First, increased mEPSC frequency onto female compared with male MSN in the rat AcbC was present prepuberty, determining that sex differences in excitatory synapse are generated before puberty and adulthood. Second, MSNs from females exposed to masculinizing levels of estradiol or testosterone during the neonatal sensitive period to organizational hormone action lacked increased mEPSC frequency compared with control females or males. This finding shows that neonatal masculinization/defeminization *via* estrogen exposure is sufficient to permanently downregulate excitatory synaptic input onto MSNs, an endocrine process which would normally occur only in male animals. This suggests an overall model that sex differences in AcbC excitatory synapse number are organized by steroid sex hormone action during early development, with estrogen exposure in males permanently decreasing AcbC synapse number and possibly adult estrogen sensitivity. Finally, Cao and colleagues found no evidence that MSN intrinsic membrane or action potential properties differed by sex, including those mediating intrinsic excitability. These findings tentatively focus the working model explaining sex differences in AcbC function toward the neuroanatomical inputs onto MSNs, most prominently glutamatergic synapse, but also dopamine inputs. Intriguing possibilities include interactions between sex, estradiol, glutamate, and dopamine. Consistent with this speculation, female compared with male AcbC MSNs demonstrate an increased proportion of large-head dendritic spines adjacent to dopaminergic terminals (87). Estradiol may also differentially modulate dopamine signaling across striatal regions, as anatomical studies revealed membrane-associated estrogen receptor α and GPER-1 expression in dopaminergic terminals in the nucleus accumbens, but not the caudate–putamen (73, 74). Dysregulation of sex differences in glutamatergic and dopaminergic signaling may potentially induce sex differences in the phenotype and incidence of AcbC-related disorders.

The anatomical source of increased excitatory input into the AcbC is unknown. One possibility of many is that the AcbC receives a sex-specific excitatory input distinct from other striatal regions. Consistent with this possibility, the AcbC receives a different set of glutamatergic inputs than other striatal regions (110, 111). These differential inputs are consistent with the AcbC mediating separate aspects of behavior than other striatal regions, including locomotor behaviors (112, 113), but also maternal, social, reward, learning, sensorimotor, and sex-related behaviors (112–121). Relatively new transgenic techniques could potentially be applied to address gaps in anatomical knowledge (122). However, inbred laboratory mice may not be effective tools to address the role of sex in modulating AcbC properties, as domestication induced female mice to lose sex- and AcbC-relevant behaviors compared with non-domesticated mice (123). In addition, many drug-abuse and other reward-mediated behaviors regulated by the nucleus accumbens are difficult or impossible to observe in mice (124). Regarding other avenues of investigation, it is unknown whether excitatory synaptic input, intrinsic excitability, and other components such as estrogen receptor α and β gene expression are modulated across estrous cycle stage. This should be a future avenue of research given the differences in striatal-mediated behaviors across the estrous and menstrual cycles. Another unknown is how GABA receptor activation and sex interact in MSNs. This question is interesting given that in select cases GABA receptor activation can facilitate MSN action potential generation (125), and that estradiol exposure can decrease extracellular GABA concentration in adult female rat caudate–putamen (126).

## No Evidence for Sex Differences in Excitatory Synaptic Input Onto MSNs in the Prepubertal AcbS and Caudate–Putamen

Prepubertal sex differences in excitatory synaptic input seem specific to the AcbC. MSNs in prepubertal male and female AcbS and caudate–putamen show no evidence of sex differences in mEPSC frequency, amplitude or decay (104, 127). Although there is no evidence of sex differences in excitatory synaptic properties so far, it is always a possibility that sex or estradiol modulates unexamined aspects of glutamatergic or other neurotransmission, including but not limited to NMDA/AMPA ratios/function, synaptic plasticity, and/or silent synapse formation. At this point it is unknown whether MSNs in adult AcbS or caudate–putamen exhibit sex differences in excitatory synaptic input, although one study used anterograde but not retrograde tracing methods to provide evidence of increased projections from the orbital frontal cortex to the caudate–putamen in adult gonad-intact female compared with male rats (128). Regarding synaptic plasticity, a recent study established that pharmacological inhibition of aromatase blocked the induction of long-term potentiation of glutamatergic excitatory inputs onto MSNs in adult gonad-intact male rat caudate–putamen neurons (81). This study did not include females, but increased estradiol concentrations in adult female caudate–putamen compared with estradiol concentrations in circulating plasma levels (129), along with the presence of aromatase in caudate–putamen and nucleus accumbens (79–82) hint that aromatase may be locally active across the striatum in both sexes.

## MSNs Show Increased Intrinsic Excitability in Female Compared with Male Prepubertal Caudate–Putamen but Not the AcbC or AcbS

Up to this point, this review has focused on sex differences in excitatory synaptic input. Alternatively, sex differences could occur in the intrinsic electrophysiological properties of MSNs, including alterations in action potential properties, the frequency of evoked action potentials to injected current properties, and passive (membrane) properties. Intrinsic electrophysiological properties regulate action potential generation in response to synaptic input (130), making these values integral in producing the functional output of MSNs. For example, augmented MSN intrinsic excitability would increase the number of action potentials that an MSN generates in response to a unit current. Alterations in MSN intrinsic excitability have been implicated in a number of striatal functions and disorders, including the responsivity to abused drugs, homeostatic plasticity, and striatal neuron subtypes (94, 131–136).

Female MSNs in prepubertal caudate–putamen show increased intrinsic excitability compared with male MSNs (127), unlike MSNs in the AcbC and AcbS (104, 109). This excitability is manifested in several respects. Female MSNs on average produce more action potentials than male MSNs for similar moderate amounts of injected positive current. This difference in action potential to current ratio is generated by an increased initial action potential firing rate in female compared with male MSNs, along with a decreased action potential after hyperpolarization peak and a mildly hyperpolarized action potential threshold. No sex differences were detected in passive membrane properties such as input resistance or in mEPSC properties. This lack of difference in mEPSC properties is essential for interpreting sex differences in MSN intrinsic excitability, as intrinsic excitability can be altered either independently or in concert with other attributes such as changes in excitatory synaptic input (94, 137–140). These findings support a model where increased excitability in prepubertal caudate–putamen MSNs is driven by internal changes to MSN electrical properties, rather than changes in excitatory synaptic input. Elucidating the ionic and receptor basis underlying this increased intrinsic excitation in female MSNs will be critical future experiments, along with assessments of adult MSNs, possible regional differences between dorsolateral and dorsomedial caudate–putamen, and the rostral–caudal axis. Changes in intrinsic excitability may be responsible for the increased excitability of female compared with male MSNs *in vivo* recorded in adult caudate–putamen (50, 69, 70). However, it is possible that intrinsic excitability and/or excitatory synaptic input in the caudate–putamen may be reorganized during puberty, such as glutamatergic synaptic input in the medial amygdala (141, 142), or striatal dopamine receptors (31, 143, 144). Either or both of these properties may be modulated by estrous cycle stage (145, 146), as suggested by *in vivo* recordings of caudate–putamen MSNs (69, 70). Overall, data from caudate–putamen MSNs support the theme of increased excitability in female compared with male MSNs, but indicate that the mechanism underlying these differences varies by striatal region.

## Conclusion

**Figure 1:**
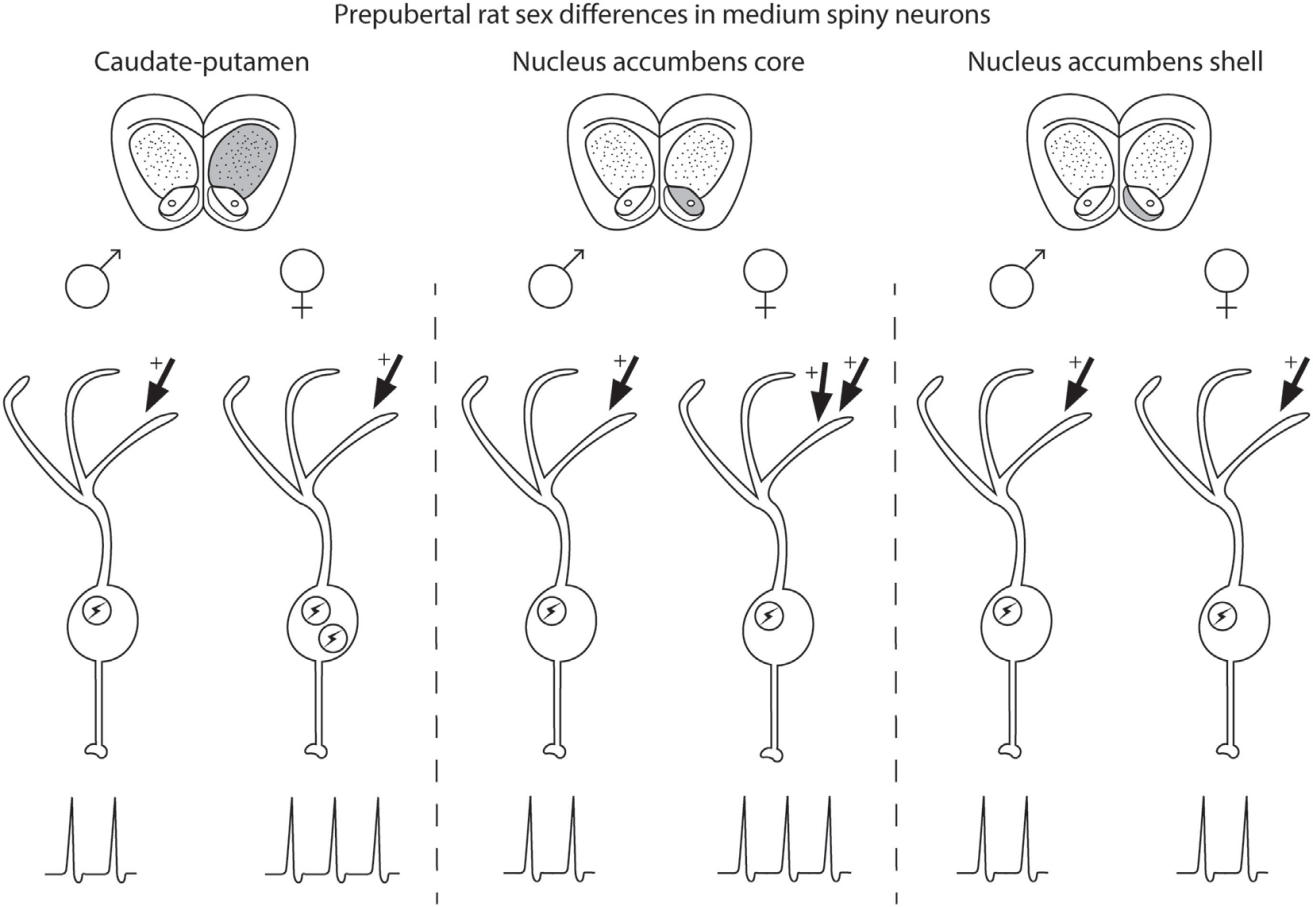
Schematic of sex differences in medium spiny neurons (MSNs) in prepubertal rat caudate–putamen, nucleus accumbens core (AcbC), and nucleus accumbens shell (AcbS). In general, MSNs recorded from prepubertal female rats exhibit increased excitation compared with male MSNs in the caudate–putamen and nucleus AcbC. However, the exact nature of the increased excitability in female MSNs differs by striatal region, encompassing changes in either intrinsic excitability (indicated in the schematic with an encircled lightning bolt) or excitatory synaptic input (indicated with arrows with plus signs). Differences in intrinsic excitability and excitatory synaptic input are indicated with more or less lightning bolts and arrows, respectively. Caudate–putamen MSNs show increased intrinsic excitability in prepubertal females compared with males. Nucleus AcbC MSNs receive augmented excitatory synaptic input in prepubertal females compared with males. Nucleus AcbS MSNs exhibit little evidence for sex differences in either intrinsic excitability or excitatory synaptic input in prepubertal animals.

Medium spiny neuron electrophysiological properties are sensitive to sex and steroid sex hormone action in a striatal region-specific manner (Figure 1). In prepubertal caudate–putamen, MSNs show on average increased intrinsic excitation in females compared with males. In adult caudate–putamen, MSNs likewise show increased excitability in females compared with males, but the mechanism underlying this difference remains unknown. In both prepubertal and adult AcbC, MSNs receive augmented excitatory synaptic input in females compared with males, and early-life exposure to estradiol is instrumental in the sexual differentiation of this property. In the AcbS, there is little evidence for sex differences in intrinsic excitability or excitatory synaptic input in prepubertal animals unexposed to adverse environmental stimuli. These data argue for heterogeneity across striatal regions in the sensitivity to MSNs to sex and steroid sex hormones and the relative amount and nature of masculinization and feminization in MSN electrophysiological properties differs by striatal region. These findings highlight the importance of differentiating between striatal regions, developmental stage, sex, and estrous cycle. These data extend earlier mosaic models of brain sexuality, in that not only individual brain regions but also individual neuron types, in this instance MSNs, show differential degrees of masculinization, feminization, and homogeny (147, 148). These sex differences and similarities in MSN electrophysiological properties, along with developmental, individual, subtype, and the other documented variables in MSN properties, induce a dizzying variety *within the same neuron type* both between and within males and females.

## Author Contributions

JM wrote the manuscript; DD prepared the figure; and JC, JW, DD, and JM edited and approved the manuscript and figure.

## Conflict of Interest Statement

The authors declare that the research was conducted in the absence of any commercial or financial relationships that could be construed as a potential conflict of interest.
